# Host antiviral factors hijack furin to block SARS-CoV-2, ebola virus, and HIV-1 glycoproteins cleavage

**DOI:** 10.1080/22221751.2022.2164742

**Published:** 2023-02-01

**Authors:** Changqing Yu, Guosheng Wang, Qiang Liu, Jingbo Zhai, Mengzhou Xue, Qiang Li, Yuanhua Xian, Chunfu Zheng

**Affiliations:** a School of Advanced Agricultural Sciences, Yibin Vocational and Technical College, Yibin, People’s Republic of China; b Institute of Animal Health, Guangdong Academy of Agricultural Sciences, Key Laboratory of Livestock Disease Prevention of Guangdong Province, Scientific Observation and Experiment Station of Veterinary Drugs and Diagnostic Techniques of Guangdong Province, Ministry of Agriculture and Rural Affairs, Guangzhou, People’s Republic of China; c Department of Pulmonary and Critical Care Medicine, Shanghai East Hospital, Tongji University School of Medicine, Shanghai, People’s Republic of China; d Nanchong Key Laboratory of Disease Prevention, Control and Detection in Livestock and Poultry, Nanchong Vocational and Technical College, Nanchong, People’s Republic of China; e Key Laboratory of Zoonose Prevention and Control at Universities of Inner Mongolia Autonomous Region, Medical College, Inner Mongolia Minzu University, Tongliao, People’s Republic of China; f Department of Cerebrovascular Diseases, The Second Affiliated Hospital of Zhengzhou University, Zhengzhou, People’s Republic of China; g Department of Microbiology, Immunology & Infection Diseases, University of Calgary, Calgary, Canada

**Keywords:** Antiviral factors, furin, viral glycoprotein, cleavage, SARS-CoV-2

## Abstract

Viral envelope glycoproteins are crucial for viral infections. In the process of enveloped viruses budding and release from the producer cells, viral envelope glycoproteins are presented on the viral membrane surface as spikes, promoting the virus's next-round infection of target cells. However, the host cells evolve counteracting mechanisms in the long-term virus-host co-evolutionary processes. For instance, the host cell antiviral factors could potently suppress viral replication by targeting their envelope glycoproteins through multiple channels, including their intracellular synthesis, glycosylation modification, assembly into virions, and binding to target cell receptors. Recently, a group of studies discovered that some host antiviral proteins specifically recognized host proprotein convertase (PC) furin and blocked its cleavage of viral envelope glycoproteins, thus impairing viral infectivity. Here, in this review, we briefly summarize several such host antiviral factors and analyze their roles in reducing furin cleavage of viral envelope glycoproteins, aiming at providing insights for future antiviral studies.

## Introduction

Viral envelope glycoproteins are a critical structural component in the viruses’ host cell infection cycles [1]. Morphologically, they are observed as typical spikes, localize on the nascent virion surface, and are responsible for recognizing cell receptors, thus inducing the merger of viral membranes and cell membranes [2]. Because of their crucial roles in cell infection, the viral envelope glycoproteins are always the targets of antivirals [3].

Currently, some studies uncovered that the host antiviral proteins also target the viral envelope glycoproteins [4]. For instance, the endoplasmic reticulum (ER)-associated protein degradation (ERAD) pathway was used to enhance the degradation of the HIV-1 envelope glycoprotein by the mitochondrial translocator protein (TSPO) [5]. Serine incorporator 5 (SERINC5) suppressed HIV-1 infection at the entry step by affecting viral envelope glycoprotein-mediated virus-target cell fusion [6,7]. However, it has been shown that the interferon (IFN)-induced transmembrane (IFITM) proteins and the lectin galactoside-binding soluble 3 binding protein (LGALS3BP/90 K) impairs the processing of viral envelope glycoproteins [8,9], suggesting that these host antiviral proteins use distinct antiviral modes.

**Figure 1.**
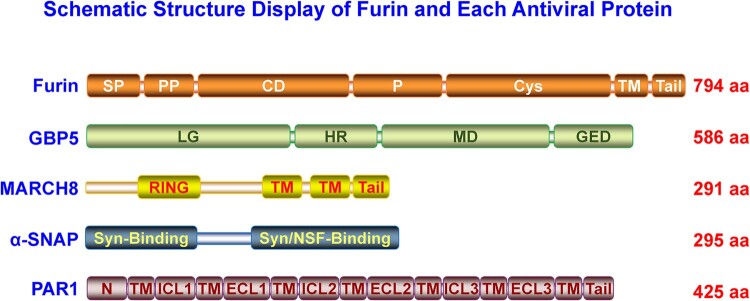
The structure analysis of furin, GBP, MARCH8, α-SNAP, and PAR1 proteins. Each specific protein domain was indicated. In furin, SP, signal peptide; PP, proprotein; CD, catalytic domain; P, P domain; Cys, cysteine-rich domain; TM, transmembrane domain; Tail, cytoplasmic tail. In GBP, LG, large GTPase domain; HR, hinge region; MD, the middle domain; GED, GTPase effector domain. In MARCH8, RING, the E3 ubiquitin ligase activity domain; TM, transmembrane domain; Tail, cytoplasmic tail. In α-SNAP, Syn-binding, syntaxin-binding region; Syn/NSF-binding, syntaxin/NSF-binding region. In PAR1, N, N-terminus region; TM, transmembrane region; ICL1/2/3, intracellular loop 1/2/3/; ECL1/2/3, extracellular loop 1/2/3.

Many intracellular proprotein convertases (PCs) could cleave class I viral envelope glycoproteins [2,10]. Furin, one member of the PC family, recognizes and cleaves many cellular proprotein substrates and invading pathogens’ components, including the viral envelope glycoproteins [10]. As shown in the schematic structure (Figure 1), furin consists of several typical PC domains, of which the catalytic domain (CD) and P domains are crucial in maintaining its PC function [11]. The P domain regulates pH, calcium dependency, and PC activity, while the CD domain is critical for substrate recognition and cleavage [11,12]. Furin is sorted into several intracellular compartments, such as ER and trans-Golgi network (TGN), involved in endosomes recycling, transported onto the plasma membrane (PM), and shed into the extracellular space via autocleaving activity, demonstrating it could cleave substrates intra- and extracellularly [11]. Recently, many studies indicated that a group of host antiviral proteins aimed at furin to block viral envelope glycoprotein cleavage. These antiviral factors include guanylate-binding proteins (GBP) 2 and 5 [13], membrane-associated Ring-CH (MARCH)8 [14], α-soluble N-ethylmaleimide sensitive fusion (NSF) attachment protein (α-SNAP) [15], and protease-activated receptor 1 (PAR1) (Figure 1) [16]. Our minireview summarizes these antiviral factors’ blocking roles and analyzes their specific antiviral mode in the following text.

## GBP2/5

GBPs belong to the family of IFN-inducible guanosine triphosphatases (GTPases) [17]. Currently, the human GBP family comprises at least 7 members and plays multiple roles in host cellular defense against pathogenic viruses, bacteria, protozoa invasion, and regulation of innate immunity signalling pathways [17].

GBPs contain a globular N-terminal large GTPase (LG) domain and a C-terminal helical domain, which are further divided into the middle domain (MD) and the GTPase effector domain (GED) (Figure 1) [18]. GTP binding could promote GBPs oligomerization and trigger their hydrolysis potency. The C-terminal CaaX isoprenylation motif and oligomerization are crucial for GBPs membrane-associated localization [19], facilitating their antiviral function.

GBP5 was demonstrated as a potential restriction factor for HIV-1 replication [20]. Subsequently, Krapp *et al.* uncovered that GBP5 impaired HIV-1 progeny virion infectivity by blocking its envelope glycoprotein cleavage, maturation, and virion incorporation and was strongly upregulated in response to IFN in monocyte-derived macrophages (MDM) and CD4^+^ T cells [21]. The follow-up study indicated that the GBP5 paralogue GBP2 also reduced HIV-1 infectivity via a similar mechanism [13].

**Figure 2.**
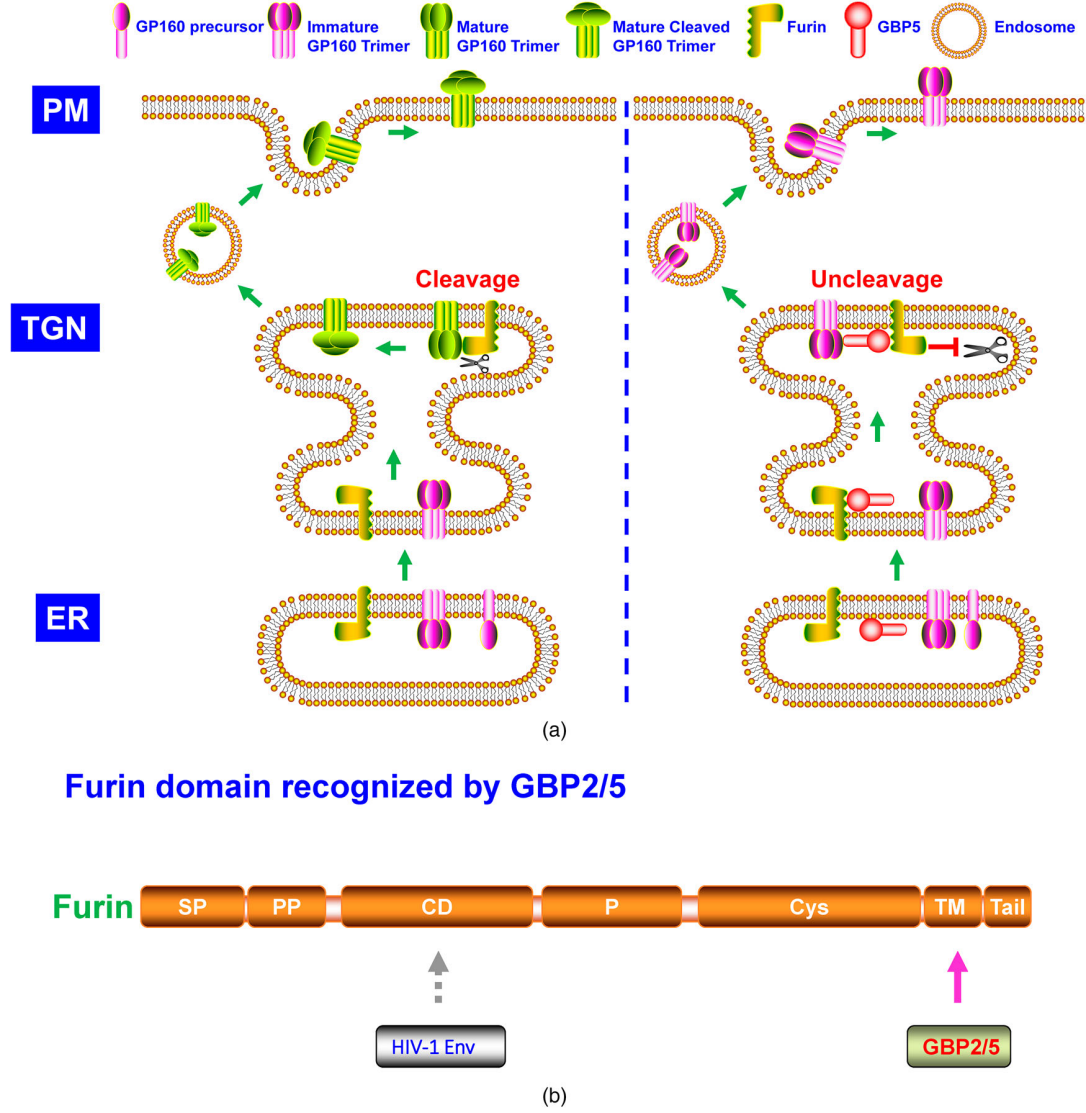
**A.** The model of GBP2/5 inhibiting furin cleavage of HIV-1 envelope (Env) glycoprotein. HIV-1 Env is high-mannose N-glycosylated in ER and then transported to the Golgi network. At TGN, HIV-1 Env (gp160) completes complex N-glycosylation modification and is processed by furin into gp120 and gp41 subunits, which are then relinked formed homologous self-trimer. The trimeric gp120-gp41 glycoproteins are subsequently transported to the plasma membrane. GBP2/5 could recognize and bind to furin at TGN and thus suppresses furin cleavage of HIV-1 gp160. The uncleaved gp160 trimer could be transported to the plasma membrane but makes the nascent virion particles less active than those containing the cleaved gp120-gp41 trimer after being integrated into the viral membranes. **B.** Furin domain recognized by GBP2/5.

In-depth work uncovered that GBP2/5 reduced HIV-1 envelope glycoprotein processing via binding to cellular protease furin and restricting its cleavage activity (Figure 2A) [13]. GBP2/5 interacted with furin and recognized its C-terminal cytoplasmic autocleavage region (Figure 2B). Notably, GBP5 but not GBP2 showed a pattern of TGN-localization. The GBP2-C588A and GBP5-C583A (C-terminal CaaX to AaaX variation) mutants lost their function in blocking HIV-1 envelope glycoprotein processing and maturation. Besides HIV-1 [13,21], GBP2/5 also inhibited furin-dependent cleavage of diverse envelope glycoproteins of viruses, including the rabies virus (RABV), European bat lyssavirus 1 (EBLV-1), Marburg virus (MARV), highly pathogenic avian influenza A virus (HPIAV), murine leukemia virus (MLV), Zika virus (ZIKV), and measles virus (MeV) [13]. Intriguingly, GBP2/5 also significantly reduced the pseudovirus infection mediated by the ancient extinct human endogenous retrovirus K (HERK) envelope glycoprotein [22], which harboured a furin-cleavage site [23]. GBP2/5 impaired HERK envelope glycoprotein cleavage and maturation [22], consistent with their blocking role on furin cleavage activity [13,21].

The newly emerged severe acute respiratory syndrome coronavirus 2 (SARS-CoV-2) spike (S) glycoprotein contains a polybasic furin-cleavage site and could be cleaved by furin [24], and the furin cleavage site in SARS-CoV-2 S protein was required for its efficient transmission in vitro and in vivo [25,26]. It thus would be interesting to determine whether GBP2/5 could effectively inhibit SARS-CoV-2 infection via blocking furin cleavage of its S glycoprotein.

Importantly, GBP2/5 also reduced infectivity of viral particles pseudotyped by Lassa virus (LASV) envelope glycoprotein, which is cleaved by another cellular protease PCSK8/SKI other than furin, suggesting the broader inhibitory effects of GBP2/5 on cellular PC [13].

Recently, Cui *et al.* dissected the structural basis of GTP-induced dimerization of GBP5 [27], which confirmed that GTP-induced oligomerization was crucial for its antiviral activity. Meanwhile, their work demonstrated that the GBP5 L307A-P308A mutant had little effect on the inhibition of furin cleavage activity and HIV-1 envelope glycoprotein processing but lowered its HIV-1 infection restriction [27], arguing that GBPs may employ furin-independent pathways to inhibit viral infection, such as altering viral envelope glycoprotein glycosylation or trafficking. The GBP2/5-furin complex crystal structure recovery is expected to provide more scientific evidence to understand GBPs’ antiviral mechanism, and whether GBP2/5-mediated furin cleavage inhibition is conserved among mammalian species remains to be elucidated.

## MARCH8

MARCH proteins were discovered because of their homology to E3 ubiquitin ligase of K3 and K5 of the Kaposi's sarcoma-associated herpesvirus (KSHV) [28]. MARCH family proteins now contain 11 members, most of which share a similar structure, i.e. N-terminal cytoplasmic C4HC3-type RING-finger domain (RING-CH finger) and two or more transmembrane domains (TM) (Figure 1), except for MARCH7 and MARCH10 without predicted TMs [29]. MARCH proteins extensively regulate cell surface adaptive immunity proteins [30], innate immunity signal transduction [31,32], and autophagy pathways [33] and are recently indicated antiviral activities [34–37].

Tada *et al.* discovered MARCH8 inhibited HIV-1 infection by reducing its envelope glycoprotein incorporation into virions [34]. Unlike vesicular stomatitis virus G-glycoprotein induced by MARCH8 to enter lysosome pathways via a mode of viral envelope glycoprotein cytoplasmic tail-dependence (CTD) [38,39], the HIV-1 envelope glycoprotein was not degraded but proved to be sequestered in intracellular compartments by MARCH8 through a mode of viral envelope glycoprotein cytoplasmic tail-independence (CTI) [38,39]. Later studies confirmed MARCH1/2 also exhibited antiviral activities [35,40], and the MARCH proteins antiviral spectrum was extended to ebolavirus (EBOV) [14,39], SARS-CoV-2 [39,40], MLV [40], influenza A virus (IAV) [41–43], spring viremia of carp virus (SVCV) [44], and many other viruses [40,45].

**Figure 3.**
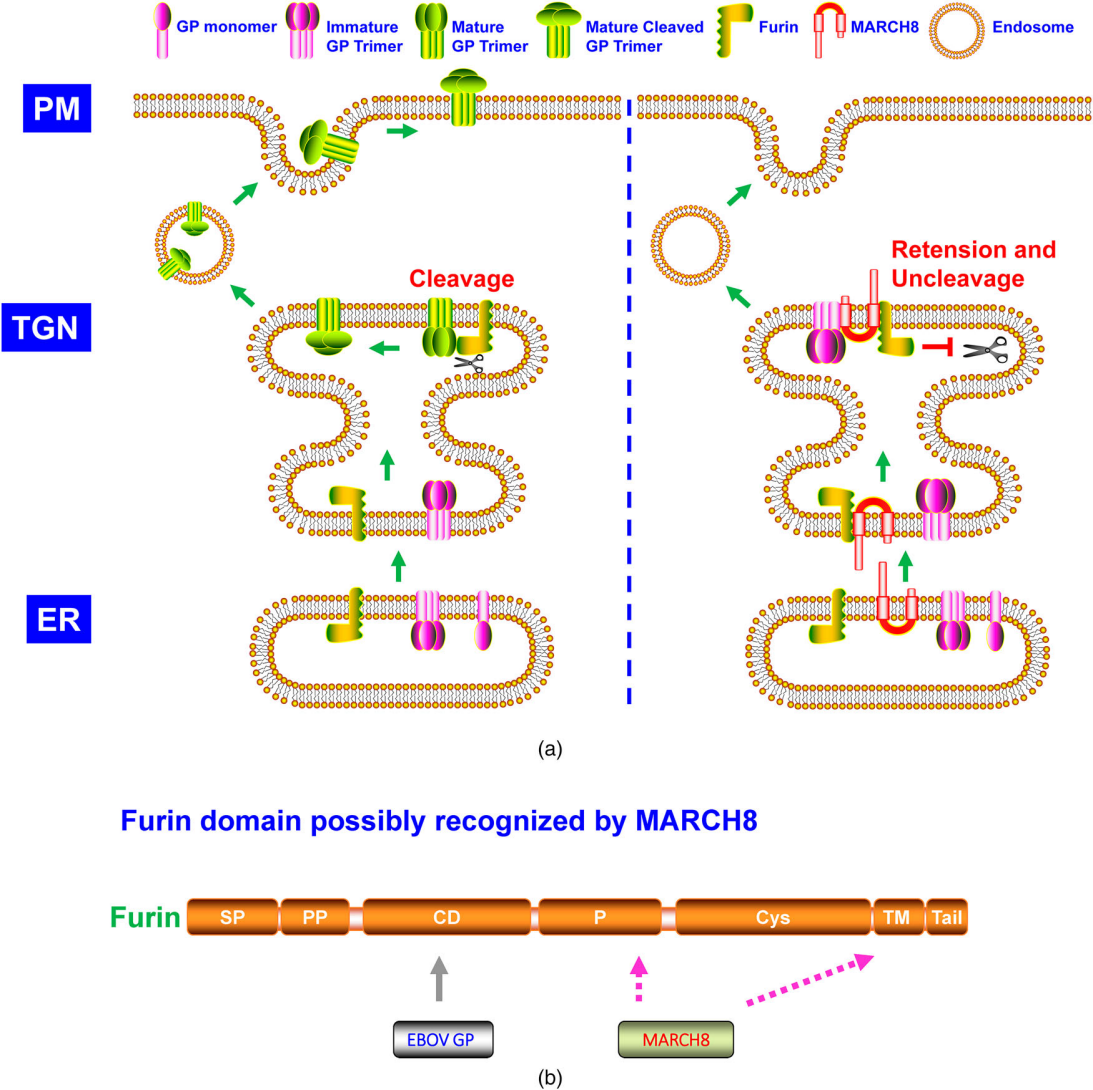
**A.** The model of MARCH8 suppresses the cleavage and glycosylation maturation of EBOV GP. In ER, EBOV GP starts high-mannose N-glycosylation. After entry into the Golgi network, EBOV GP experiences complex N-glycosylation and O- glycosylation and is cleaved into two GP subunits (GP1 and GP2) by host furin proprotein convertase. The GP1 and GP2 relink and form the self-trimer, which is then transported to the cell surface. However, at TGN, in the presence of MARCH8, furin cleavage activity is blocked. Correspondingly, EBOV GP is unable to be cleaved by furin. In addition, MARCH8 traps the EBOV GP at TGN, leading to its retention and thus blocking its further transportation to the cell plasma membrane. PM: plasma membrane; TGN: trans-Golgi network; ER: endoplasmic reticulum. **B.** Furin domain recognized by MARCH8.

**Figure 4.**
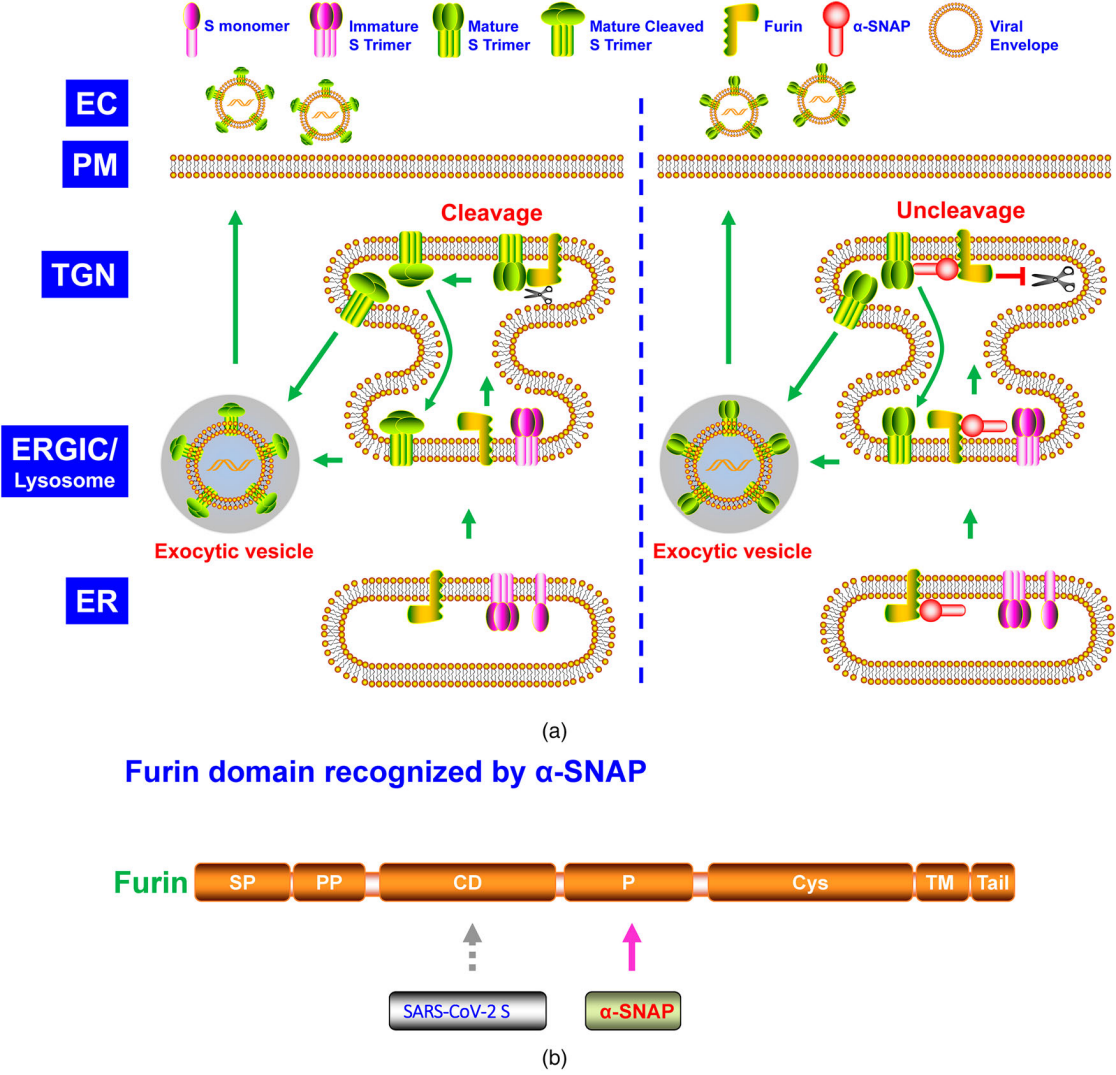
**A.** The model of α-SNAP suppressing the cleavage of SARS-CoV-2 Sipke (S) glycoprotein. SARS-CoV-2 S glycoprotein is synthesized, high-mannose N-glycosylated, and trimerized in ER, where it is transported to the Golgi network. The S trimer is modified with complex N-glycosylation and O- glycosylation, cleaving into S1 and S2 subunits by furin. The S1-S2 trimer is transported to the ER-Golgi intermediate compartment (ERGIC) through retrograde trafficking or lysosome, where the virus assembly and egress happen. The S glycoprotein cleavage is blocked in the presence of α-SNAP at TGN. When integrated into the SARS-CoV-2 virion particles, the virion with uncleaved S glycoprotein is less infectious than that with cleaved S1-S2 glycoprotein. **B.** Furin domain recognized by α-SNAP. EC, extracellular space.

Under the CTI antiviral pattern, MARCH8 was identified to block EBOV glycoproteins (GP) cleavage and glycosylation maturation (Figure 3A) [14,39]. MARCH8 interacted with EBOV GP and furin, and EBOV GP bound to furin CD, demonstrating MARCH8 recognized furin beyond its CD (Figure 3B) [14]. The EBOV GP-furin complex was preserved in TGN but not ER in the presence of MARCH8 (Figure 3A) [14]. Previously, it was reported that MARCH2 proteins could induce redistribution of transporter proteins, including syntaxin 6 and SNAP receptors (SNAREs) that mediated endosomal trafficking [46]. Similarly, the retention of the EBOV GP-furin complex at TGN by MARCH8 may result from the translocation of these transporter machinery proteins. A critical intracellular membrane trafficking regulation protein, α-SNAP, inhibited SARS-CoV-2 infection by blocking furin-mediated S glycoprotein cleavage (Figure 4A) [15]. It thus should be necessary to explore whether MARCH8 retained the EBOV GP-furin complex via interference of α-SNAP trafficking. Lun *et al.* observed MARCH8's blocking effects on SARS-CoV-2 S glycoprotein cleavage and glycosylation maturation [39], similar to the observed phenotype in EBOV GP. Whether SARS-CoV-2 S glycoprotein inefficient cleavage arose from MARCH8 blocking of furin cleavage activity needs to be clarified.

MARCH8 has high lung expression and restricted IAV H1N1 infection *in vitro* and *in vivo* [41,42]. It thus deserves to verify MARCH8 anti-SARS-CoV-2 infection in vivo. Notably and intriguingly, different from the CTI antiviral mode [38,39], it was found MARCH8 also engaged the CTD antiviral pattern in reducing HIV-1 infection [40]. Therefore, it would be intriguing to determine whether MARCH8 combats the same virus infection via both fruin-dependent (CTI) and furin-independent (CTD) antiviral mechanisms.

It was reported that MARCH1/2 used the CTI antiviral mode similar to MARCH8 in restricting HIV-1 infection [35]. It is necessary to determine whether MARCH1/2 inhibits furin cleavage of the HIV-1 envelope glycoprotein and whether MARCH members from other mammalian species can use the furin-targeted CTI antiviral mechanism. The MARCH8-furin complex crystal structure has yet to be acquired, and its future dissection will provide more detailed information for MARCH8 antiviral effects.

## α-SNAP

SNAP family proteins in mammals contain three isoforms, i.e. the α, β, and γ-SNAP, which are involved in cellular vesicle trafficking machinery [47]. α- and γ-SNAP showed a ubiquitous expression pattern in many tissues, while β-SNAP indicated a specific expression in the brain [48]. α-SNAP has a more than 80% amino acid (aa) homology with β-SNAP while sharing about 25% identity with γ-SNAP [48]. All SNAPs contain several predicted coiled-coil structures implying for mediation of protein interaction (Figure 1). The NSF/α-SNAP/SNARE complex crystal structure was dissected [49,50].

Generally, α-SNAP binds to its membrane-associated receptor SNARE proteins, then recruits the NSF and forms a heterooligomeric complex [51]. NSF-mediated ATP hydrolysis induced SNARE conformational changes and promoted the disassembly of the heterooligomeric complex [52].

Over the past years, few studies have investigated the α-SNAP antiviral function. Recently, α-SNAP was demonstrated to inhibit SARS-CoV-2 infection *in vitro* by impairing its cellular entry (Figure 4A) [15]. SARS-CoV-2 S glycoprotein contained a furin cleavage site between the S1 and S2 subunit. α-SNAP bound to furin and blocked its cleavage on SARS-CoV-2 S glycoprotein, and the α-SNAP N-terminal 40 aa and C-terminus 231–250 aa were critical for its furin recognition [15].

Like GBP2/5 [13], α-SNAP is also IFN-inducible [15]. Differed from GBP2/5 and possibly MARCH8 [13,14], the P domain of furin was necessary for α-SNAP recognition (Figure 4B) [15]. β-SNAP but not γ-SNAP showed a similar pattern to α-SNAP on inhibition of SARS-CoV-2 S glycoprotein cleavage, probably due to the high aa sequence identity between α-SNAP and β-SNAP [15]. Other than SARS-CoV-2, α-SNAP also blocked the envelope glycoprotein cleavage of EBOV, MARV, and MERS-CoV, designating a relatively broad spectrum of α-SNAP antiviral function [15].

The NSF/α-SNAP/SNARE complex is important in intracellular vesicle trafficking. However, Wang *et al.* demonstrated that the C-terminal 45 aa of α-SNAP (Figure 1), which interacts with NSF, was not required for blocking SARS-CoV-2 S glycoprotein cleavage [15], confirming a novel and vesicle trafficking-independent mode of α-SNAP biological function. Notably, their work also indicated that the α-SNAP F27S/F28S mutant weakened its furin binding but blocked SARS-CoV-2 infection early, implying an additional antiviral mode, independent of inhibition of SARS-CoV-2 S glycoprotein cleavage, which deserves further investigation.

## PAR1

The PAR family proteins belong to the G-protein coupled receptor (GPCR) superfamily and currently include four members termed PAR1-4 [53]. Structurally, PAR proteins contain an extracellular N-terminus, including the signal peptide, seven TM helices, three intracellular loops, three extracellular loops (ECL), and an intracellular C-terminus (Figure 1) [54]. PAR1 and PAR2 were activated via proteolytic cleavage of their N-terminal propeptide to expose the tethered ligand (TI), which interacted with their ECL domain and thus induced conformational changes that can elicit intracellular signal transduction [54]. PAR1 and PAR2 are localized at intracellular membrane compartments such as TGN or PM and are widely involved in signalling pathways, which play multiple roles in inflammation, cancer, and embryogenesis [55].

**Figure 5.**
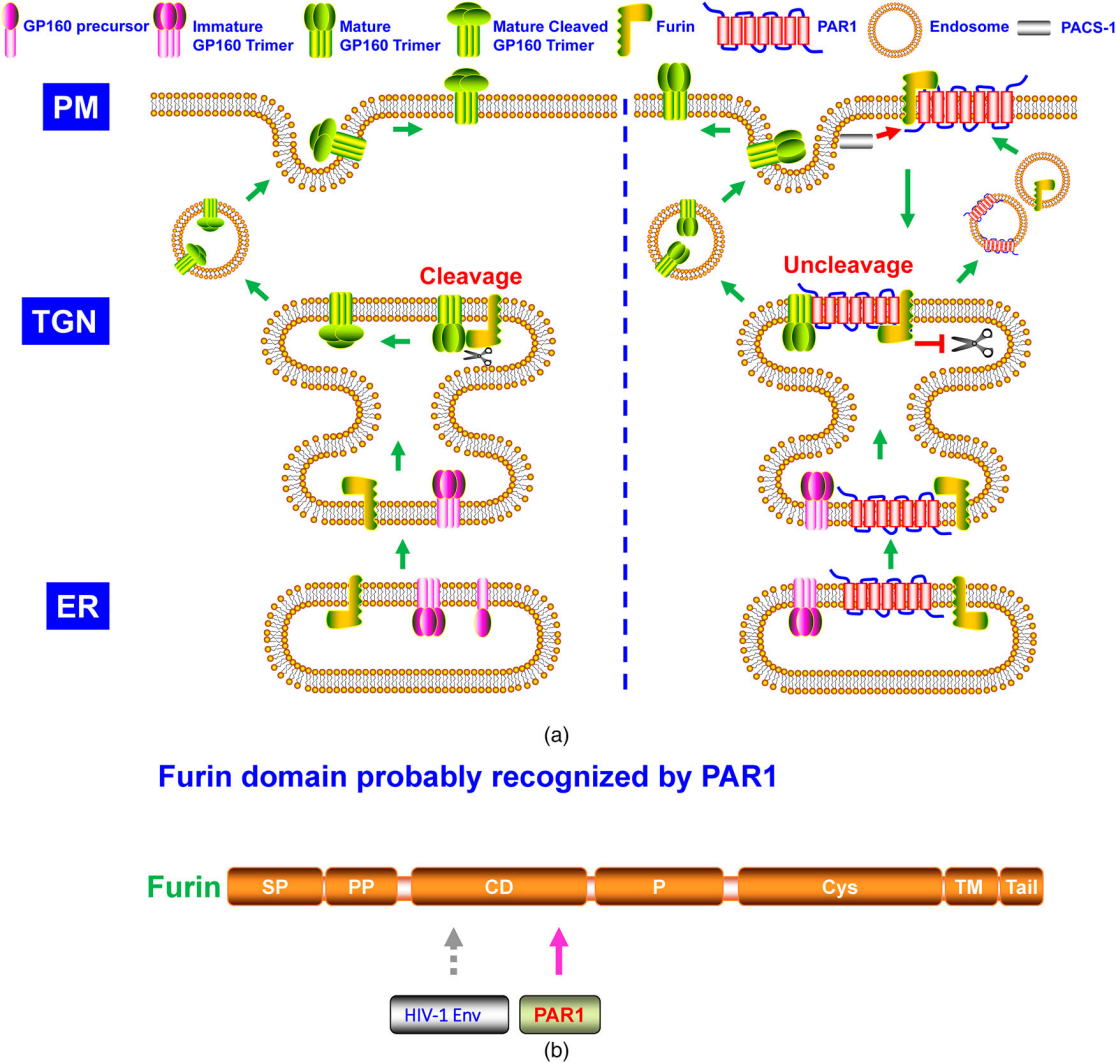
**A.** The model of PAR1 suppressing the cleavage of HIV-1 glycoprotein. As described in Figure 2, HIV-1 gp160 needs to be cleaved by furin. PAR1 interacts with furin, which is downregulated by PACS-1 from the plasma membrane and trapped at TGN, where furin is inhibited from cleaving HIV-1 gp160. **B.** Furin domain recognized by PAR1.

Aerts *et al.* discovered that PAR1 antagonist reduced symptomatic human metapneumovirus (hMPV) infection in mice, and PAR1 inhibited furin cleavage of hMPV F protein [56]. Subsequent studies indicated that PAR1 also suppressed HIV-1 gp160 cleavage by furin (Figure 5A) [16]. PAR1 could be cleaved by soluble PC5A and paired basic amino acid cleaving enzyme 4 (PACE4) at R46. Although identified by the two proteases, membrane-bound furin and PC5B did not cleave the cell surface PAR1. Further study showed PAR1-furin complex at PM was translocated to and trapped at TGN [16]. Cytosolic adapter phosphofurin acidic cluster sorting protein 1 (PACS1) played a key role in PAR1-furin complex cell surface downregulation [57]. Both cytoplasmic tails of furin and PAR1 could be targeted by PACS1 in their PM translocation. Binding by PAR1 made furin unable to cleave its viral envelope substrates and blocked viral replication (Figure 5A) [57]. N-terminal TI of PAR1 (PR_41_SFLLR_46_N), but not the PAR1 (PR_41_SFLLA_46_N) mutant, blocked furin cleavage of HIV-1 gp160, indicating the region contained the two basic residues (R_41_ and R_46_) was recognized by furin [16] probably via its CD domain (Figure 5B), which was responsible for furin substrate cleavage. Because of mutual antagonization, the PAR1-furin complex and their translocation provided a protective mechanism in HIV-associated neurocognitive disorders (HAND) [57].

Similar to GBP2/5, MARCH8, and α-SNAP blocking role on viral infection by targeting furin [13–15], it is rational to deduce that PAR1 probably restricts infection of a broad spectrum of enveloped viruses, other than hMPV and HIV-1 [16,56,57], whose envelope glycoproteins depend on furin cleavage. Meantime, it deserves to try whether PAR1-mediated viral infection inhibition is conserved across mammalian species and whether PAR1 played roles in HIV-1 or viruses’ cross-species transmission.

## LGALS3BP/90 K, IFITMs, and inhibitors

LGALS3BP/90 K is a secreted IFN-inducible glycoprotein and has multiple roles in neoplastic transformation and innate immunity [58]. Originally, it was demonstrated that 90 K was upregulated in HIV-1-infected CD4^+^ T cells and individuals [59]. Lodermeyer *et al.* dissected the anti-HIV-1 infection role of 90 K [9], and it was subsequently confirmed that 90k inhibited the replication of various kinds of viruses via activating the innate immune signalling [60]. Overexpression of 90 K in 293 T cells reduced HIV-1 infection, while endogenous knockdown of 90 K in HeLa cells and primary macrophages enhanced HIV-1 infection; IFN-α stimulation upregulated cellular 90 K expression in 293 T cells and macrophages and enhanced its anti-HIV-1 infection activity [9].

HIV-1 gp160 proteolytic processing and gp120 virion incorporation were impaired in the presence of 90 K [9]. To verify whether furin was involved in 90K-mediated inhibition of HIV-1 gp160 cleavage, several known furin substrates were tested, including EBOV GP, IAV H7 hemagglutinin (HA), and GPC-3. However, EBOV GP cleavage was significantly blocked in the presence of 90 K, while IAV HA0 and cellular GPC-3 precursors cleavage were less affected by 90 K [9]. Thus, the authors demonstrated that furin was less possibly targeted by 90 K. Interestingly, though impaired HIV-1 gp160 cleavage, 90 K did not directly interact with HIV-1 envelope glycoproteins [9]. A direct binding assay was needed between furin and 90 K but was not performed in their work. Follow-up work showed that the antiviral activity of the 90 K was conserved across primates except for rhesus macaque [61]. In addition, besides blocking HIV-1 envelope glycoprotein processing, 90 K was also demonstrated to inhibit HIV-1 virion production in a vimentin filaments-dependent manner [62].

Recently, an investigation indicated LGALS3BP/90 K was markedly elevated in COVID-19 patients [63], a phenomenon similarly observed in AIDS patients [59]. However, in contrast to 90K's unable to bind to HIV-1 envelope glycoprotein [9], SARS-CoV-2 spike glycoprotein pulled down LGALS3BP/90 K, and the LGALS3BP/90 K overexpression inhibited SARS-CoV-2 spike-pseudotyped viral particles entry and spike-induced cell–cell fusion in vitro [63], indicating 90 K impaired SARS-CoV-2 S glycoprotein function. Considering SARS-CoV-2 S contained the furin cleavage site [24], 90 K may recognize and interfere furin cleavage of SARS-CoV-2 S. As in the case of 90 K restricting HIV-1 infection [9], a direct binding verification is also needed amongst LGALS3BP/90 K, furin, and SARS-CoV-2 S in future work. IFITMs are membrane-associated and ubiquitously expressed proteins that have multiple roles in cellular innate and adaptive immunity [64,65]. The human IFITM family includes 5 members, IFITM1-3, IFITM-5, and IFITM-10 [66]. Brass *et al.* discovered that IFITM proteins restricted replication of IAV H1N1, West Nile virus (WNV), and dengue virus (DENV) [8]. Subsequent studies showed that IFITM broadly restricted a group of viruses, including EBOV, MARV, HIV-1, ZIKV, SARS-CoV-1, and SARS-CoV-2 [66–68]. Compton *et al.* reported that IFITM1-3 restricted HIV-1 cell-to-cell infection [69], and Yu *et al*. found that IFITM2/3 substantially reduced HIV-1 envelope glycoprotein processing [70]. As mentioned above, furin could cleave HIV-1 gp160. Therefore, IFITM may interact with furin to impair HIV-1 gp160 proteolytic processing, which needs further examination. Interestingly, while limiting SARS-CoV-2 replication [68], SARS-CoV-2 occasionally hijacked IFITMs to increase viral infection [71,72].

In addition to host antiviral factors, small molecular compounds targeting furin are also used for clinical antiviral strategies [10]. The well-known decanoyl-Arg-Val-Lys-Arg-chloromethyl ketone (dec-RVKR-cmk, CMK), a small synthetic inhibitor of PC including furin, suppressed cleavage of HIV-1 envelope glycoprotein, ZIKV prM, and SARS-CoV-2 S [73–75]. Some furin inhibitors significantly reduced viral titres of WNV and DENV and the spread of IAV [76,77]. Obatoclax was reported to inhibit the replication of IAV, ZIKV, and SARS-CoV-2 [78–80], and it utilized multiple strategies to block SARS-CoV-2 infection, including reducing furin expressions [80].

Statins, the cholesterol-lowering drug, was confirmed to suppress HIV-1 and EBOV infection [81,82]. It reduced EBOV GP cleavage, glycosylation modification, and GP incorporation into virions, similar to the MARCH8 modulation effects on EBOV GP [82]. Though not demonstrated previously, it deserves to try whether statins target furin to block viral replication.

## Concluding remarks

Here, we summarized recently identified host innate immunity factors that exerted their antiviral activities through inhibiting furin cleavage activity, including GBP2/5, MARCH8, α-SNAP, PAR1, possibly LGALS3BP/90 K and IFITMs, and some inhibitors/drugs. Their antiviral spectrum can be extended from SARS-CoV-2, EBOV, and HIV-1 to many other enveloped viruses. Generally, they all could block the cleavage of viral envelope glycoproteins. However, besides cleavage inhibition, GBP2/5 and MARCH8 also used distinct modes to block viral replication, including altering the envelope glycoprotein glycosylation modification or its intracellular trafficking, which indicated a complex regulation of their antiviral strategies. GBP2/5 and α-SNAP were IFN-inducible [13,15], while MARCH8 was not sensitive and showed highly endogenous expressions [34], indicating a solid intrinsic immunity defense against virus infection.

Though these antiviral factors potently inhibited virus replication, fewer viral antagonizing mechanisms were reported. HIV-1 evaded GBP5 by mutations of Vpu, which improved HIV-1 Env production and, thus, more resistant to GBP5 [21]. IAV H1N1 could evade MARCH8 restriction via M2 K78R mutation [41]. MLV glycoGag could neutralize IFITM3 and thus enhance viral replication [83]. Therefore, it is necessary to determine whether other viruses also evolved such resisting mechanisms. Notably, furin as cellular PC is responsible for the cleavage of cellular proteins, implying these antiviral factors are also involved in regulating cellular proproteins cleavage.

In conclusion, furin is targeted by many host proteins to block viral infection. Future studies are warranted to explore more novel host antiviral factors, as discussed here, and the mechanism dissection of such antiviral factors will shed new light on antiviral drugs and vaccine design and development.

## Author contributions

C.Y., G.W., and Q.L. contributed to the concept of this work, images acquisition, and manuscript preparation; J.Z. contributed to images acquisition; M.X., Q.L., Y.X. and C.Z. contributed to the concept of this work, images acquisition, and manuscript preparation and revision. All authors contributed to this study and approved the submitted version.

## Data Availability

The data supporting this study's findings are available upon reasonable request.
